# Human Papillomavirus Genotype Richness and the Biodiversity of Squamous and Glandular Cervical Dysplasias: A Cross-Sectional Study

**DOI:** 10.3390/pathogens12101234

**Published:** 2023-10-11

**Authors:** Elisa Gozzini, Davide Radice, Fabio Bottari, Sara Boveri, Maria Elena Guerrieri, Eleonora Petra Preti, Noemi Spolti, Mariacristina Ghioni, Federico Ferrari, Anna Daniela Iacobone

**Affiliations:** 1 Department of Clinical and Experimental Sciences, University of Brescia, 25136 Brescia, Italy; e.gozzini92@gmail.com; 2 Preventive Gynecology Unit, European Institute of Oncology IRCCS, 20141 Milan, Italy; mariaelena.guerrieri@ieo.it (M.E.G.); eleonora.preti@ieo.it (E.P.P.); noemi.spolti@ieo.it (N.S.); annadaniela.iacobone@ieo.it (A.D.I.); 3 Division of Epidemiology and Biostatistics, European Institute of Oncology IRCCS, 20141 Milan, Italy; davide.radice@ieo.it; 4 Division of Laboratory Medicine, European Institute of Oncology IRCCS, 20141 Milan, Italy; fabio.bottari@ieo.it; 5 Laboratory of Biostatistics and Data Management, Scientific Directorate, IRCCS Policlinico San Donato, San Donato Milanese, 20097 Milan, Italy; sara.boveri@grupposandonato.it; 6 Division of Pathology, European Institute of Oncology IRCCS, 20141 Milan, Italy; mariacristina.ghioni@ieo.it

**Keywords:** human papillomavirus (HPV), multiple infections, HPV genotype richness, biodiversity, cervical dysplasia, squamous lesions, glandular lesions

## Abstract

The impact of multiple infections on the risk of cervical lesions is a subject of ongoing debate. This study aims to explore whether the richness of HPV genotype infections and the biodiversity of squamous and glandular cervical dysplasias could influence the progression of precancerous lesions. We conducted a cross-sectional analysis involving 469 women who attended the Colposcopy Unit at the European Institute of Oncology in Milan, Italy, from December 2006 to December 2014. HPV type richness was measured as the number of different genotypes per patient. We calculated the associations between richness and age, as well as histologic grade, along with Simpson’s biodiversity index for cervical dysplasias. We observed significant inverse relationships between the richness of high-risk (HR) genotypes and both age (*p* = 0.007) and histologic grade (*p* < 0.001). Furthermore, as the histologic grade increased, the mean biodiversity index of cervical dysplasias decreased, with exceptions noted in cases of normal histology and adenocarcinoma in situ. Different histologic grades formed five clusters with distinct mean ages and mean biodiversity indices. These findings suggest that HPV genotype richness and the biodiversity of cervical dysplasias may play a crucial role in predicting the risk of high-grade cervical lesions, enabling personalized management of precancers.

## 1. Introduction

Human papillomaviruses (HPVs) constitute a family composed of more than 150 genotypes capable of infecting both cutaneous and mucosal epithelium [1]. Among these, the most clinically relevant are 14 alphapapillomavirus genotypes defined as high-risk (HR), as they are epidemiologically associated with cervical carcinoma and are found in 99.7% of tumors [2]. For this reason, HR-HPV genotype infection is considered a necessary but not sufficient cause for the development of cervical carcinoma [3]. In the majority of cases, especially in women under 30 years of age, HPV infections progress silently and resolve spontaneously [4]. In their systematic review, Bonde et al. emphasized that the persistence of the same HR-HPV type is the most significant risk factor for the development of high-grade preneoplastic lesions and cervical carcinoma. Furthermore, among HR-HPV genotypes, not all are equally capable of leading to the development of cervical intraepithelial neoplasia (CIN) grade 2 or higher [5]. Particularly in high-grade intraepithelial lesions (HSILs), the presence of HPV 16, 18, 31, or 45 carries a higher risk of progression to cervical carcinoma than other HR genotypes [6,7].

In general, the likelihood of acquiring an HPV infection is highest in the age group ranging from 20 to 24 years (27.4%) and gradually decreases with increasing age, stabilizing around 19% among those aged 50 to 59 years [8]. Additionally, the likelihood of having multiple infections is highest in the population under 30 years of age and gradually decreases with age [9]. In populations of women with cervical cancer who have not undergone primary or secondary prevention, approximately 50% acquire HPV infection at around 20.6 years of age, 75% at 30.6 years, and only 10% after the age of 40 [10]. These considerations, along with an understanding of the pathogenetic process from cervical mucosal infection to the development of neoplastic pathology, have led to the adoption of HR-HPV testing as the basis for screening for this type of cancer.

Currently, the impact of multiple infections on the risk of cervical lesions is debated, as are their occurrence by chance or as a consequence of various interactions and risk factors, including age, smoking, sexual habits, and immunosuppression [11,12,13]. Several studies have demonstrated that infection by multiple HPV genotypes constitutes a significant risk factor for the development of high-grade cervical lesions (CIN2+) [9,14,15,16,17,18,19,20,21]. Moreover, these reports conclude that multiple infections not only correlate with the severity of cervical dysplasia but also synergistically contribute to the persistence of HPV infection and the subsequent carcinogenic process. Conversely, other studies highlight that the majority of high-grade dysplasias and cervical tumors are instead driven by a single HR-HPV genotype, whereas multiple infections are observed in young patients with low-grade intraepithelial lesions (LSILs) or negative cytology [9,11,20,22,23,24]. Schmitt et al. analyzed the impact of viral load assessed in dysplastic lesions, concluding that multiple infections with a high viral load are more commonly found in LSILs (62.6%) compared with HSILs (51.9%) and negative cervical biopsies (19%). However, the presence of multiple infections alone does not differ between high- and low-grade cervical lesions [25]. In their review, Zhou et al. conclude that viral load cannot be used as the only biomarker of cervical dysplasia progression, and it should be combined with other methods in order to maximize its prognostic value [26].

In ecology, biological diversity expresses the variety within an ecosystem and is characterized by two parameters, namely “richness”, which is the number of different species in the same sample, and “evenness”, which measures the relative abundance of individual species within the same ecosystem [27,28]. In the context of the ongoing debate concerning the oncogenic role of single versus multiple infections, the objective of the present study is the analysis of whether the richness of HPV genotype infections and the biodiversity of cervical dysplasias may play a significant role in determining the progression of precancerous lesions. We also aim to ascertain whether they represent substantial risk factors that should be taken into account when assessing women with persistent HR-HPV infections, particularly in relation to squamous and glandular cervical lesions.

## 2. Materials and Methods

### 2.1. Population

All women attending the Colposcopy Unit at the Preventive Gynecology Unit of the European Institute of Oncology IRCCS, Milan, Italy, between December 2006 and December 2014, were identified from hospital file archives and selected for cross-sectional analysis. The local institutional review board approved the study protocol (protocol IEO S544/210, date of approval: 26 May 2010). Written informed consent was obtained from all subjects prior to colposcopy or surgical treatment for the use of data for scientific purposes. Patients were included if they met the following criteria: (a) age at diagnosis of 18 years or older; (b) colposcopic-guided cervical biopsies due to abnormal Pap smear or surgical treatment for preneoplastic and neoplastic cervical lesions; (c) histological confirmation of any grade of CIN, adenocarcinoma in situ (AIS), and invasive cervical carcinoma (ICC); and (d) known HPV status. Patients were excluded if they (a) denied informed consent or (b) had unavailable HPV status. Data regarding the main clinical, laboratory, and pathological characteristics of patients were recorded in a dedicated database. All histological diagnoses were conducted on colposcopic-guided biopsies (at least two punches of the transformation zone alone or with endocervical curettage) or on surgical tissue by dedicated gynecological pathologists from the Division of Pathology at our Institute.

### 2.2. Human Papillomavirus DNA Detection and Genotyping

A ThinPrep PreservCyt cervical sample (Hologic, Inc., Bedford, MA, USA) was collected from all patients to perform a Linear Array HPV Genotyping Test (Roche Diagnostics, Pleasanton, CA, USA). The linear array test uses biotinylated PGMY09/11 consensus primers to amplify a 450 bp region of the L1 gene, detecting the following 37 HPV genotypes: HPV 6, 11, 16, 18, 26, 31, 33, 35, 39, 40, 42, 45, 51, 52, 53, 54, 55, 56, 58, 59, 61, 62, 64, 66, 67, 68, 69, 70, 71, 72, 73 (MM9), 81, 82 (MM4), 83 (MM7), 84 (MM8), IS39, and CP6108. The denatured polymerase chain reaction products were then hybridized to an array strip containing immobilized oligonucleotide probes. The results were visually interpreted by two independent operators using the provided reference guide, following the manufacturer’s protocol, and subsequently compared to reach a final conclusion.

### 2.3. Statistical Analysis

HPV type richness is defined as the number of different genotypes per patient and is categorized as 0, 1, or ≥2. The Simpson’s biodiversity index (1 − *D_k_*) is calculated according to the following formula:

(1) $$1-D_{k}=\sum_{j=1}^{S_{k}}\frac{n_{kj}\left(n_{kj}-1\right)}{N_{k}\left(N_{k}-1\right)}$$

where *n_kj_*, *N_k_*, and *S_k_* are the abundance of the *j*-th HPV type, the total abundance of each species, and the number of species for the *k*-th histologic grade, respectively. As such (1 − *D_k_*) ranges from 0 (no biodiversity) to 1 (infinite biodiversity). The histologic grade was categorized on *k* = 4 levels as Normal, CIN1, CIN2, and CIN3+, where CIN3+ was defined as having a CIN3 grade or AIS or ICC. Age parameters included mean, standard deviation (SD), median, and interquartile range (IQR) and were tabulated using HR and low-risk (LR) richness as well as histologic grade. The significance of the association between age and richness or the histologic grade was assessed using the Kruskal–Wallis test, and the association between richness and histologic grade was determined using the chi-square test. Empirical Simpson’s biodiversity index for single HR type infection distributions was obtained using the nonparametric bootstrap, stratified via histologic grade resampling with replacement with 10,000 replications. Bootstrap 95% confidence intervals (95% CIs) were estimated using the percentile method. HPV types 16, 18, 31, 33, 35, 39, 45, 51, 52, 56, 58, 59, 66, and 68 were considered high-risk (HR) while all other genotypes identified through the linear array test were classified as low-risk (LR) types. All tests were two-tailed and considered significant at the 5% level. All analyses were carried out using SAS 9.4 (SAS Institute Inc., Cary, NC, USA) and Stata (StataCorp. 2023. Stata Statistical Software (Release 18), College Station, TX, USA, StataCorp LLC).

## 3. Results

After applying the inclusion and exclusion criteria, the final study population consisted of 469 women with a mean age of 38.9 ± 9.1 years (range: 22–83 years). All subjects were unvaccinated at the baseline.

The most common histologic grade was CIN3+, observed in 266 patients (56.7%), including 215 women diagnosed with CIN3, 39 with ICC, and 12 with AIS. In total, 76 patients (16.2%) did not test positive for any HR types, while 335 patients (71.4%) did not show any LR types. The most prevalent infection was a single HR type, accounting for 321 patients (68.4%), irrespective of the presence of any LR type. Two or more types were less common for both HR and LR, with 72 patients (15.4%) and 51 patients (10.9%) having multiple infections, respectively (see Table 1).

**Figure 1:**
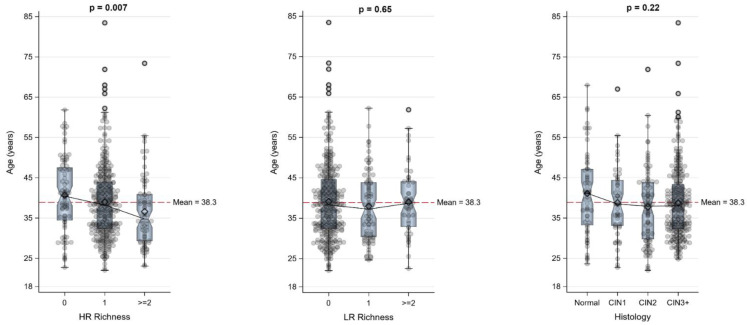
Age distribution relative to high- and low-risk genotype richness and histology.

The median age for patients with no HR infection was 40.5 years, while the median age for patients with single or multiple HR infections was 38.3 and 34.8 years, respectively. This inverse relationship between HR genotype richness and age was statistically significant (*p* = 0.007). There was no significant association between age and LR richness (*p* = 0.65) or age and histologic grade (*p* = 0.22) (refer to Table 1 and Figure 1).

**Figure 2:**
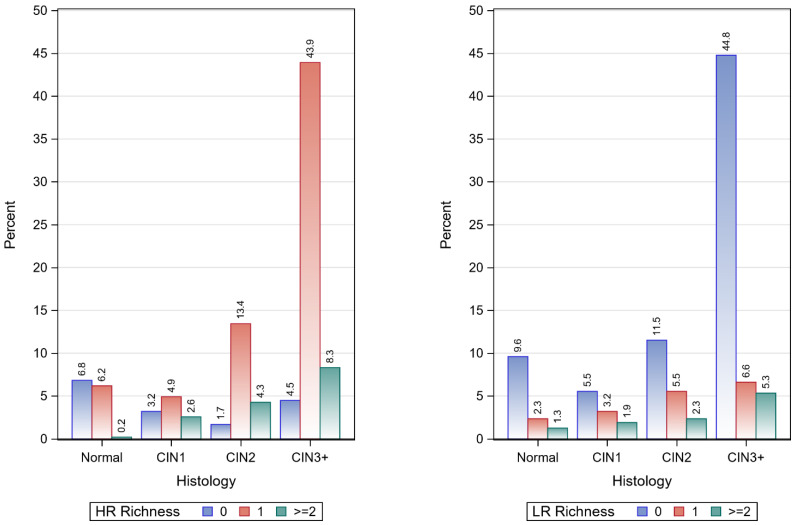
Frequency distribution of high- and low-risk genotype richness by histology.

A significant association (*p* < 0.001) was observed between both HR and LR richness and histologic grade. The richness of HR types decreased as the histologic grade increased; notably, 43.4% of patients with CIN3+ had only one HR type, compared with the lower histologic grades (see Table 2 and Figure 2).

There was a significant association between HR and LR type richness for normal histologic grades (*p* = 0.04) and CIN2 grades (*p* = 0.004) but not for CIN1 (*p* = 0.79) and CIN3+ (*p* = 0.68) (see Table 3).

**Figure 3:**
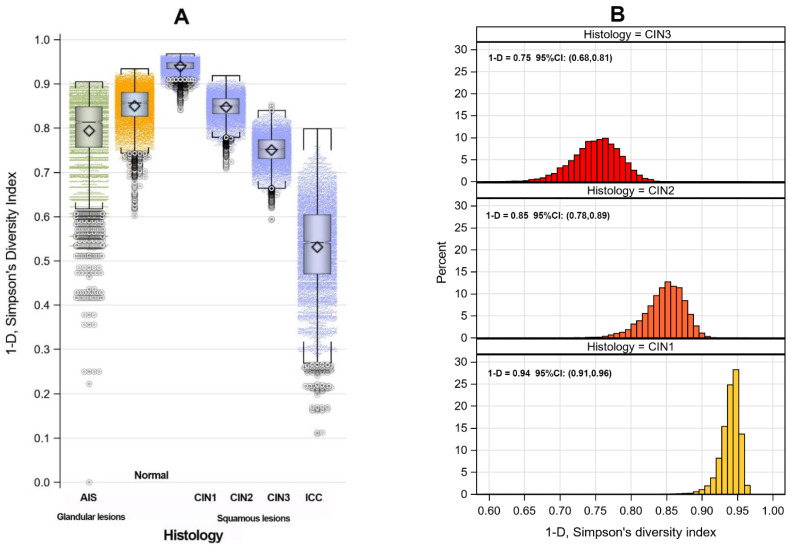
Simpson’s biodiversity index empirical bootstrap distributions box-plot (**A**) and (**B**) histograms with average estimates and 95% Confidence Intervals (CI) by histology.

Empirical bootstrap distributions for single HR-type infections demonstrated that, as the histologic grade increased, the mean biodiversity index decreased. The normal and AIS grades were exceptions, with mean biodiversity indices of 1 − D = 0.85 (95% CI: 0.75, 0.91) and 1 − D = 0.79 (95% CI: 0.59, 0.89), respectively. The mean biodiversity indices for CIN1, CIN2, CIN3, and ICC were as follows: 1 − D = 0.94 (95% CI: 0.91, 0.96); 1 − D = 0.85 (95% CI: 0.78, 0.89); 1 − D = 0.75 (95% CI: 0.68, 0.81); and 1 − D = 0.53 (95% CI: 0.30, 0.70), respectively (Figure 3A,B).

**Figure 4:**
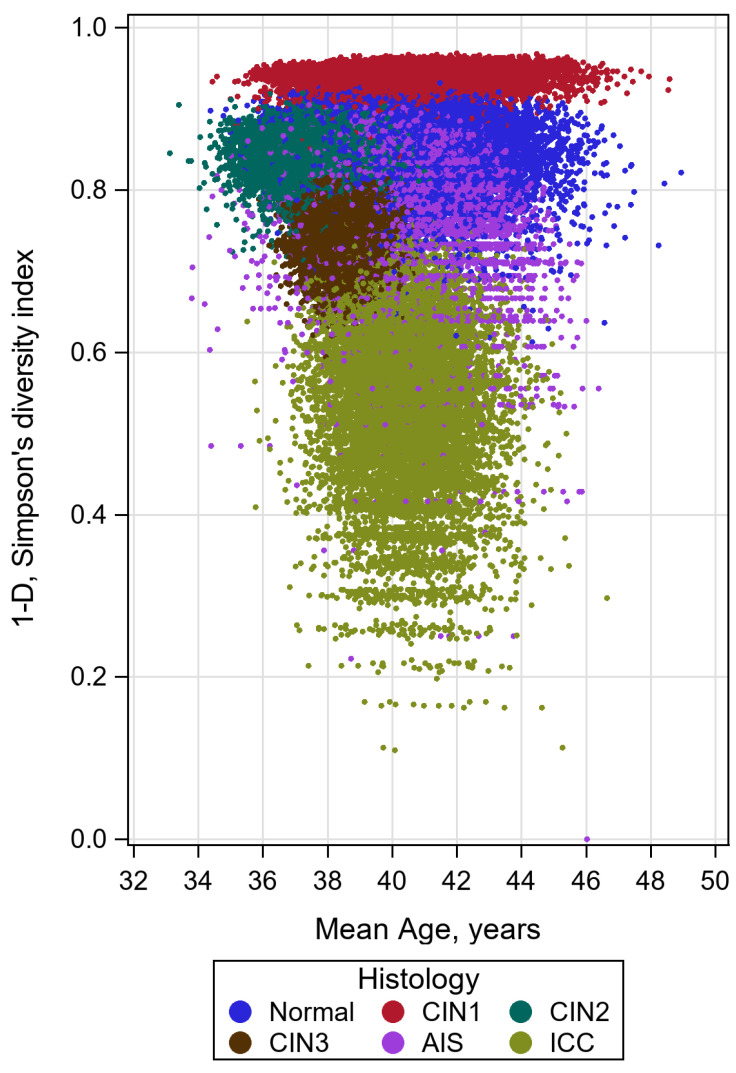
High-risk single infections biodiversity index by age.

Different histologic grades formed five clusters with distinct mean ages and mean biodiversity indices. Although there was considerable overlap between all clusters, the CIN1 grade exhibited the widest range in age and the lowest in biodiversity index, whereas ICC was the histologic grade with the greatest spread in biodiversity index and the lowest in age (see Figure 4).

Specific HPV genotype frequency distribution, HPV genotype frequency distribution needle plots by histology for HR Richness, HPV genotype frequency distribution needle plots by histology for HR Richness ≥ 2, HPV genotype frequency distribution needle plots by histology for LR Richness = 1 and HPV genotype frequency distribution needle plots by histology for LR Richness ≥ 2 are detailed in Supplementary Materials.

## 4. Discussion

The results of this study reveal a significant correlation between HR-HPV richness and the histological grade of cervical dysplasia. This association is particularly evident in squamous lesions, where there is a discernible gradient. As the histological grade of the lesion increases, biodiversity decreases. Notably, 43% of patients with CIN3+ lesions exhibited only one HR-HPV type. The principle “one virus–one lesion” aligns consistently with findings in the existing research [29,30,31,32]. It also corresponds to the pathogenetic process of cervical carcinoma, in which HR-HPV integrates its genome and drives cellular transformation toward neoplastic development [6,29,33].

Detecting the presence of HPV in histological samples is more accurate than in cervical samples, as demonstrated by several studies in the literature. Within the landscape of existing tests, the linear array test targets L1 viral protein rather than E6/E7 oncogenes; during the oncogenic process, HR-HPV viruses may integrate their genomes into cellular DNA, and during this process, the virus can lose the L1 gene, making the test no longer capable of detecting the presence of the virus. Beyond this assumption, linear array performance has been validated by the VALGENT framework, showing similar sensitivity with higher specificity to detect CIN2+ than the Hibryd Capture 2 test, and the detection of 13 hrHPV types fulfills the clinical accuracy requirements for primary cervical cancer screening [34]. Quint et al. [35] demonstrated that by genotyping HPVs within dysplastic lesion samples obtained through laser capture micro-dissection (LCM-PCR), in the case of CIN3 lesions with multiple viral infections, only one HPV type was predominant in high-grade dysplastic areas, while low-grade CIN lesions in the same biopsy block could also be attributed to other HPV types. Our study did not uncover a significant correlation between age and histological grade. However, other studies in the literature emphasize that the age group with the highest prevalence of cervical HSIL is between 31 and 40 years, while LSILs are more common in younger women [36]. This difference arises due to the study’s design, which is cross-sectional rather than longitudinal. Nevertheless, these data are continually evolving, as some evidence suggests that anti-HPV vaccination has had positive effects in reducing the prevalence of cervical HSIL in vaccinated cohorts of women, especially among younger patients [37].

When analyzing the correlation between age and HPV infection, it becomes evident that multiple genotype infections, both HR-HPV and LR-HPV, are more frequent in younger age groups. Specifically, women with multiple HR-HPV infections are, on average, four years younger than women with multiple LR-HPV infections, as shown in Table 1. This finding aligns with previous evidence in the literature: Cuschieri et al. found that multiple infections from both HR-HPV and LR-HPV are more common among women aged <30 years and become less frequent in women aged >55 years [23]. However, contrary to some authors’ findings that HR-HPV and LR-HPV infections have a second peak among postmenopausal women [18,38], this pattern did not emerge in our study, possibly due to the cross-sectional design.

Significantly, we observed an association between both HR and LR richness and histologic grade. The richness of LR and HR types decreased as the histologic grade increased. This is in agreement with previous studies indicating that single HR-HPV infections become significantly more common as the cervical lesion grade worsens. Additionally, multiple genotype infections are associated with a lower risk of CIN2+ [39,40]. Li et al. also found that a single HPV 16 infection is linked to a higher incidence of CIN2+ compared to multiple HPV 16 infections [40]. Other authors have confirmed that multiple infections versus single infections do not confer any additional significant risk for CIN2+ across all HR-HPV genotypes, regardless of HPV 16 [41]. Furthermore, recent studies have suggested that the identification of specific HPV genotypes and normalized viral load, but not the presence of multiple HPV infections, may serve as potential biomarkers for identifying women at an increased risk of progression to cervical cancer [24]. In fact, serial type-specific viral load measurements could aid in distinguishing persistent and progressing HPV infections from regressing infections in women infected with multiple HPV genotypes. This distinction is important, as it is well established that clonal reproduction and an increase in viral load of a single HPV type occur in women with multiple infections that ultimately develop CIN3+ [42].

In contrast, according to Spinillo et al., multiple HR-HPV infections are associated with larger cervical lesions, especially in women infected by HPV 16 and in CIN3+ lesions. Larger lesions are linearly associated with increasing severity of CIN [43]. Likewise, in a retrospective cohort analysis of patients who underwent conization without prior histological proof of HSIL, Wittenborn et al. showed that the detection of multiple HR-HPV infections and major colposcopic findings predict the presence of CIN2+ in diagnostic loop excisions [44]. Surprisingly, Zhong et al. found that specific HPV genotypes, such as HPV52, 53, 56, 51, 39, 66, 59, 68, and 35, are more likely to develop CIN2+ in multiple infections than in single infections, as opposed to HPV 16, suggesting that interaction with other genotypes enhances their pathogenicity [45].

As already found by Herrero et al. [18], our analysis demonstrated a significant association between HR-HPV and LR-HPV richness in CIN2 histological grades, with almost 50% of CIN2 showing a single HR-HPV infection without an association of LR genotypes. A similar association was found in normal histological grades but seemed to be a random finding due to the small number of cases, as indicated by the *p*-value (*p* = 0.04). However, this correlation, although not statistically significant, is also apparent in the CIN3+ category. This absence of a statistically significant association can be interpreted by specifying that the CIN3+ category is highly heterogeneous, encompassing not only CIN3 but also AIS and squamous ICC.

Nevertheless, it is now widely accepted that CIN2 represents a different category from CIN3+, which warrants distinct and individualized management. Recent Bayesian analyses have separately reported the risk of CIN2 and CIN3+ based on individual HR-HPV genotypes, supporting the concept that CIN2 is distinct in histological and biological identity from CIN3+ [44,46]. Indeed, up to 60% of CIN2 lesions may spontaneously regress, especially in young, non-smoking, and nulliparous women. This suggests that active surveillance, rather than immediate treatment, might be suitable for this subgroup of patients to avoid adverse obstetric outcomes related to surgery [47,48]. Several risk factors, including cytology, p16 immunohistochemistry, HPV genotyping, and DNA methylation, have been investigated to better define personalized conservative management for women concerned about treatment effects. Looking for reliable predictors of persistence and progression, Bruno et al. showed that multiple HPV genotype infections are associated with a higher rate of histological regression (96.6%) and a lower rate of histological and viral persistence (3.4%) in CIN2 when compared to single infections (61.8% and 20.3%, respectively), with no proven cases of progression [49].

Importantly, as the histologic grade increases, the mean biodiversity index decreases for single HR-HPV infections. The CIN1 category represents the group of lesions with the highest biodiversity index. This finding is consistent with the concept that cervical LSIL represents the clinical and morphological expression of active replicating HPV infection, without a significant risk of developing invasive neoplasia, as reiterated by the WHO classification of female genital tumors [50]. Moreover, as the biodiversity index decreases, the variability of genotypes responsible for squamous cervical preneoplastic lesions increases. Although CIN2+ lesions are mainly caused by single HPV infections, different HR genotypes contribute to these preneoplastic squamous lesions, not limited to genotypes 16 and 18. As previously shown in large cohort studies, HPV 16, 31, 33, 52, and 58 are most frequently detected in CIN 2–3 lesions, especially in single infections, but their hierarchy varies by geographical area. HPV 16 and 18 still remain the most prevalent genotypes in ICC worldwide [39,51,52,53]. Furthermore, in another larger cohort study of ours, we found that HPV 33, 39, and 45 are the only genotypes statistically related to the increasing number of genotypes per infection but not to worsening histology. In contrast, the single HPV 16 infection is the only one significantly correlated with a higher risk of CIN2+ [39].

Interestingly, the group with AIS histology exhibited a higher biodiversity index than the high-grade squamous lesions, although still lower than the group of HPV-infected patients without histologically confirmed lesions. This finding could be attributed to the lower number of AIS cases in our study sample and the fact that glandular dysplasias originate as such, rather than progressing through degrees as in cervical squamous intraepithelial lesions. No data are available in the previous literature regarding differences in the distribution of HPV genotype richness and biodiversity index between glandular and squamous cervical dysplasias. Li et al. only reported a similar gradient in the distribution of HR-HPV genotype richness in squamous precancerous and cancerous cervical lesions, finding a significant increase in the proportion of single HPV genotype infections as the cervical lesion worsens from CIN1 to squamous ICC [40].

Lastly, a distinct distribution of histologic grade categories was identified according to age and biodiversity index. In particular, the CIN1 grade exhibited the widest range in age and the lowest range in biodiversity index. In contrast, ICC showed the greatest variation in biodiversity index and the lowest age range. Actually, it is well recognized that CIN1 solely represents the biological expression of HPV infection and not a pretumoral lesion. Therefore, it can occur at any age. In contrast, cervical carcinoma results from persistent integrated HPV infection and a prolonged transformation process of the cervical epithelium, with the peak incidence of ICC occurring after the age of 40 years.

To our knowledge, this is the first study to apply the statistical concept of biodiversity, as measured by Simpson’s index, to cervical lesions rather than the genome of HPV genotypes. The difference between our study and other work published in the literature is that we investigated the concept of biodiversity related to the presence of various HPV genotypes, both HR-HVP and LR-HPV, present in dysplastic lesions of different histological grades. This approach allowed us to investigate the role of single versus multiple infections in cervical carcinogenesis and the differences in biodiversity between squamous and glandular cervical dysplasias. The strength of our study lies in the availability of histological diagnoses performed by experienced gynecologic pathologists for all enrolled women, ensuring the reliability and reproducibility of the results. Nevertheless, limitations of this study include selection bias related to the single-center analysis, a cross-sectional study design, and a relatively small representation of multiple infections for both HR and LR types.

## 5. Conclusions

HPV genotype richness and biodiversity of cervical dysplasias may play a fundamental role in predicting the risk of high-grade cervical lesions and allowing for the individualized management of potentially regressive precancers, especially CIN2 in young women with multiple genotype infections. However, this correlation was not demonstrated for glandular cervical dysplasias, as their origin does not follow a progressive degree pattern as seen in squamous lesions. Further longitudinal studies with larger, multicenter cohorts are needed to confirm the impact of single versus multiple genotype infections and the biodiversity index of cervical dysplasias in the development of precancerous and cancerous lesions of the cervix in the context of persistent HPV infections.

## Figures and Tables

**Table 1:** Summary statistics for age (years) by histology and HPV type richness (N= 469).

		Statistics			
		Mean (SD)	Median	IQR	*p*-Value
HR Richness					
0	N = 76	40.7 (9.3)	40.5	34,48	
1	N = 321	39.0 (9.0)	38.3	32,44	
≥2	N = 72	36.6 (9.0)	34.8	29,41	0.007
LR Richness					
0	N = 335	39.1 (9.4)	38.3	32,45	
1	N = 83	37.9 (8.5)	37.3	30,44	
≥2	N = 51	39.0 (8.1)	38.7	33,44	0.65
Histology					
Normal	N = 62	41.1 (10.4)	41.2	33,47	
CIN1	N = 50	38.8 (8.8)	38.5	33,44	
CIN2	N = 91	37.8 (9.2)	38.0	30,44	
CIN3+	N = 266	38.8 (8.7)	38.0	32,43	0.22

HR = high risk; LR = low risk; CIN = cervical intraepithelial neoplasia; SD = standard deviation; IQR = interquartile range.

**Table 2:** Frequency distribution of high- and low-risk genotype richness based on histology.

		Histology, N (%)				
	All Patients N = 469	Normal N = 62	CIN1 N = 50	CIN2 N = 91	CIN3+ N = 266	*p*-Value
HR Richness						
0	76 (16.2)	32 (6.8)	15 (3.2)	8 (1.7)	21 (4.5)	
1	321 (68.4)	29 (6.2)	23 (4.9)	63 (13.4)	206 (43.9)	
≥2	72 (15.4)	1 (0.2)	12 (2.6)	20 (4.3)	39 (8.3)	<0.001
LR Richness						
0	335 (71.4)	45 (9.6)	26 (5.5)	54 (11.5)	210 (44.8)	
1	83 (17.7)	11 (2.4)	15 (3.2)	26 (5.5)	31 (6.6)	
≥2	51 (10.9)	6 (1.3)	9 (1.9)	11 (2.4)	25 (5.3)	<0.001

HR = high risk; LR = low risk; CIN = cervical intraepithelial neoplasia.

**Table 3:** High- and low-risk genotype richness association based on histology.

			LR Richness, N (%)			
Histology	HR Richness	Row Total (%)	0	1	≥2	*p*-Value
Normal	0	32 (53.1)	26 (41.9)	2 (3.2)	4 (6.5)	
	1	29 (45.3)	19 (30.7)	8 (12.9)	2 (3.2)	
	≥2	1 (1.6)	0	1 (1.6)	0	0.04
CIN1	0	15 (30.0)	8 (16.0)	5 (10.0)	2 (4.0)	
	1	23 (46.0)	13 (26.0)	5 (10.0)	5 (10.0)	
	≥2	12 (24.0)	5 (10.0)	5 (10.0)	2 (4.0)	0.79
CIN2	0	8 (8.8)	3 (3.3)	3 (3.3)	2 (2.2)	
	1	63 (69.2)	45 (49.5)	14 (15.4)	4 (4.4)	
	≥2	20 (22.0)	6 (6.6)	9 (9.9)	5 (5.5)	0.004
CIN3+	0	21 (7.9)	16 (6.0)	2 (0.8)	3 (1.1)	
	1	206 (77.4)	163 (61.3)	26 (9.8)	17 (6.4)	
	≥2	39 (14.7)	31 (11.7)	3 (1.1)	5 (1.9)	0.68

HR = high risk; LR = low risk; CIN = cervical intraepithelial neoplasia.

## Data Availability

The data presented in this study are available upon request from the corresponding author. The data are not publicly available due to patients’ privacy restrictions. The data are safely stored in a private database of the European Institute of Oncology, Milan, Italy.

## Supplementary Materials

The following supporting information can be downloaded at: https://www.mdpi.com/article/10.3390/pathogens12101234/s1, Figure S1: HPV genotype frequency distribution needle plots by histology for HR Richness = 1; Figure S2: HPV genotype frequency distribution needle plots by histology for HR Richness ≥ 2; Figure S3: HPV genotype frequency distribution needle plots by histology for LR Richness = 1; Figure S4: HPV genotype frequency distribution needle plots by histology for LR Richness ≥ 2.
