# Supplementary material for: Human Papillomavirus Genotype Richness and the Biodiversity of Squamous and Glandular Cervical Dysplasias: A Cross-Sectional Study
Source: Pathogens. 2023 Oct 11;12(10):1234. doi: 10.3390/pathogens12101234 (PMC10610312; doi:10.3390/pathogens12101234)
Supplement: Supplementary file 1 [file pathogens-12-01234-s001.zip › pathogens-2601843-supplementary.pdf]

Figures: Fig.S.1. to Fig.S.4 show the needle plots for the frequency distribution of the detected viral genotypes according to HR- and LR-richness analyses by histological classification. The genotype identifcation number is showen above each needle. Since denominators that are too small would lead to unstable and unrepresentative estimates of relative frequencies in the general population, each plot shows the distribution only for samples whose size is not less than 10. That is the reason why Fig.S.2. do not show the normal (Fig.S.2) and Fig.S.4. the normal and CIN1 histological classifications respectively (see Table 2 for reference). The two most prevalent genotypes for each cross-classification were as follow: HPV-16 (48%), and HPV-45 (10%), HPV-16 (17%) and HPV-31 (17%), HPV-16 (41%) and HPV-31 (27%), HPV-16 (64%) and HPV-31 (11%) for the normal, CIN1, CIN2 and CIN3+ respectively (HR Richness = 1, Fig.S.1); HPV-16 (33%) and HPV-59 (33%), HPV-16 (80%) and HPV-51 (35%), HPV-16 (74%) and HPV-31 (23%) for CIN1,CIN2 and CIN3+ respcetively (HR Richness $\geq$ 2, Fig.S.2); HPV-16 (45%) and HPV-81 (27%), HPV-42 (27%) and HPV-53 (27%), HPV-16 (50%) and HPV-31 (19%), HPV-16 (52%) and HPV-18 (19%) for Normal, CIN1,CIN2 and CIN3+ respectively (LR Richness = 1, Fig.S.3); HPV-61 (55%) and HPV-16 (37%), HPV-16 (52%) and HPV-61 (44%) for CIN2 and CIN3+ respectively (Fig.S.4)

Figure S1. HPV genotypes frequency distribution needle plots by histology for HR Richness = 1

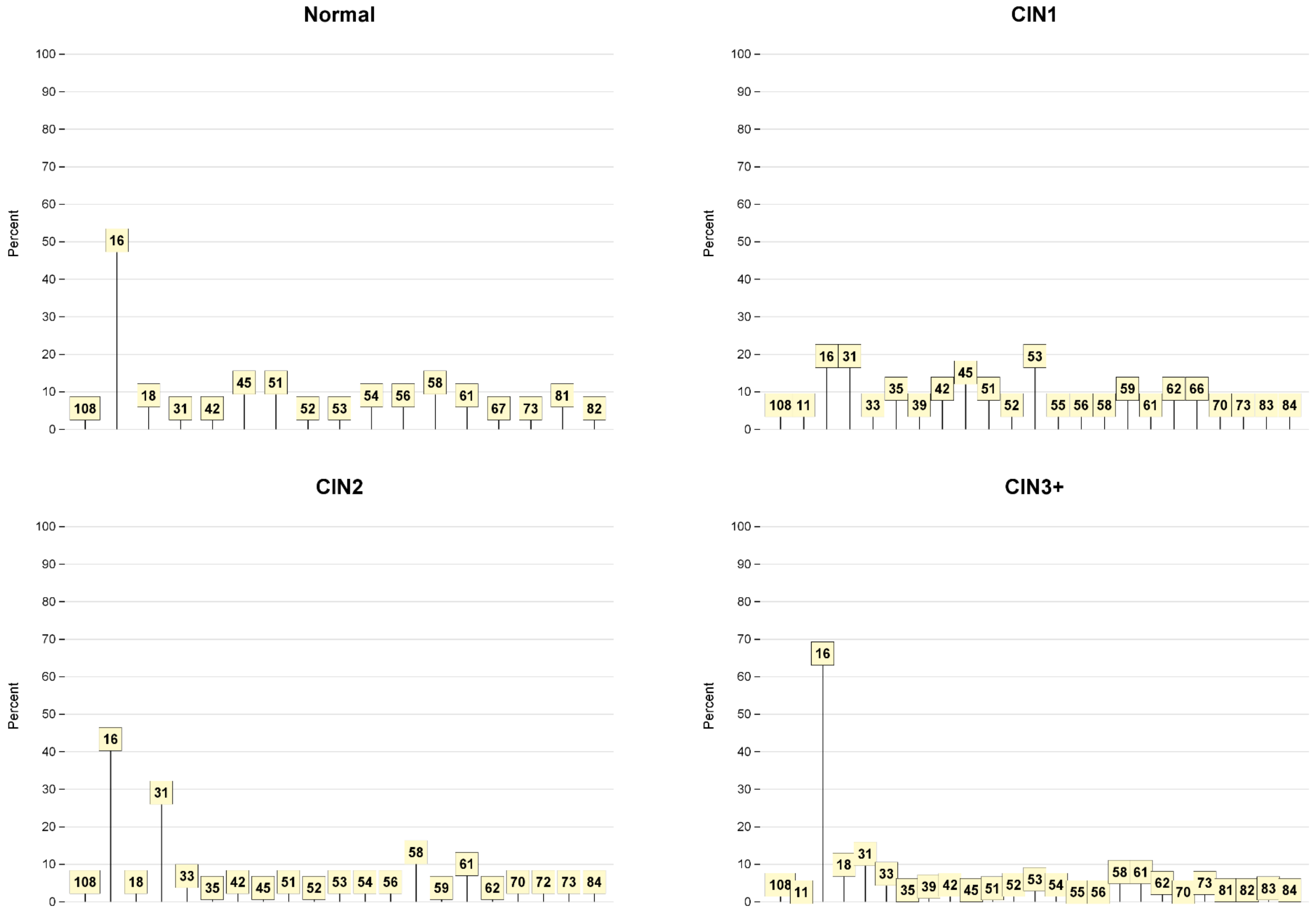

Within square numbers identify the genotype, e.g.: 16 = HPV 16, 108 = HPV CP6108 etc; Minimun Sample Size N = 10;

Figure S2. HPV genotypes frequency distribution needle plots by histology for HR Richness ≥ 2

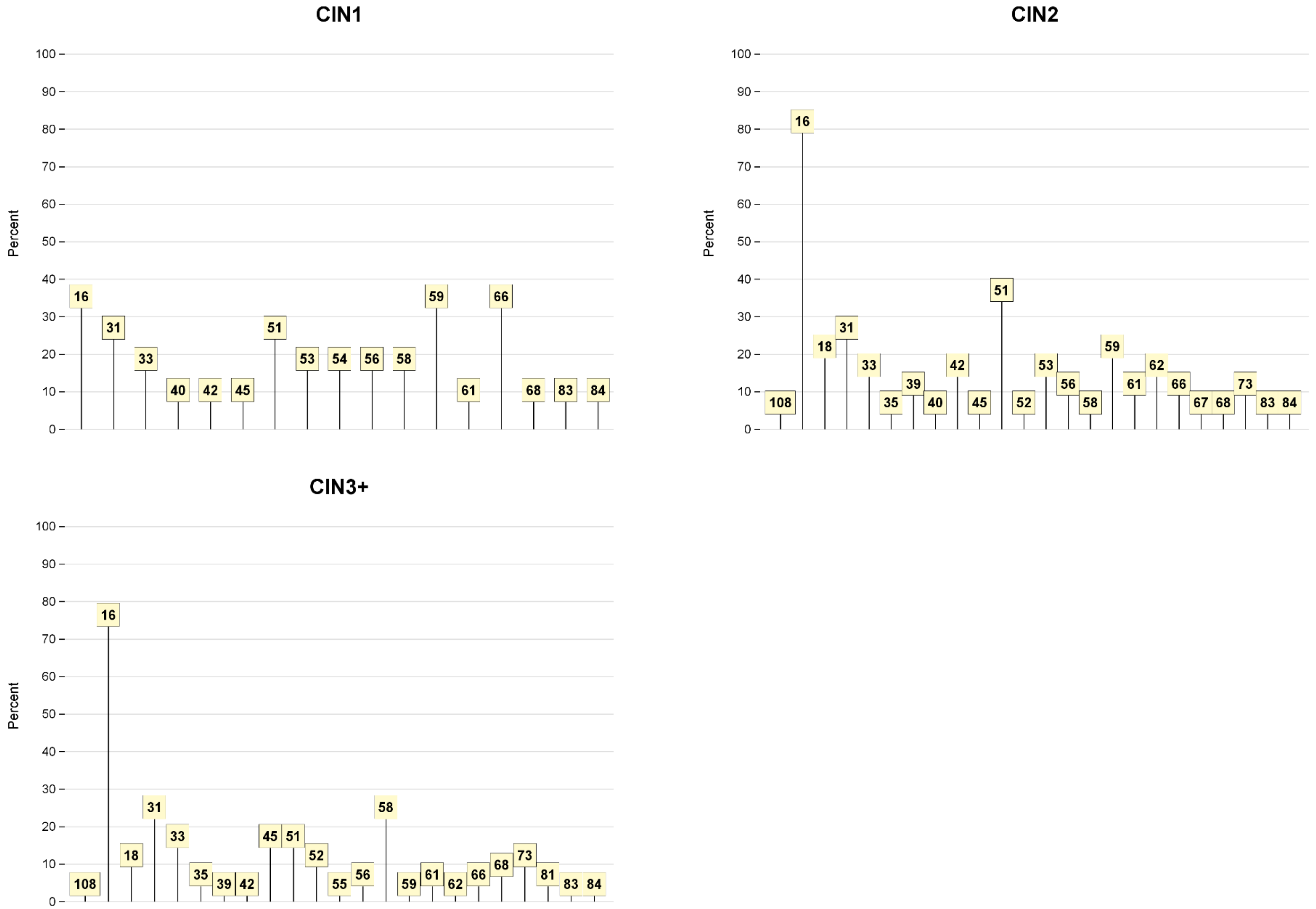

Within square numbers identify the genotype, 16 = HPV 16, 108 = HPV CP6108 etc; Minimun Sample Size N = 10;

Figure S3. HPV genotypes frequency distribution needle plots by histology for LR Richness = 1

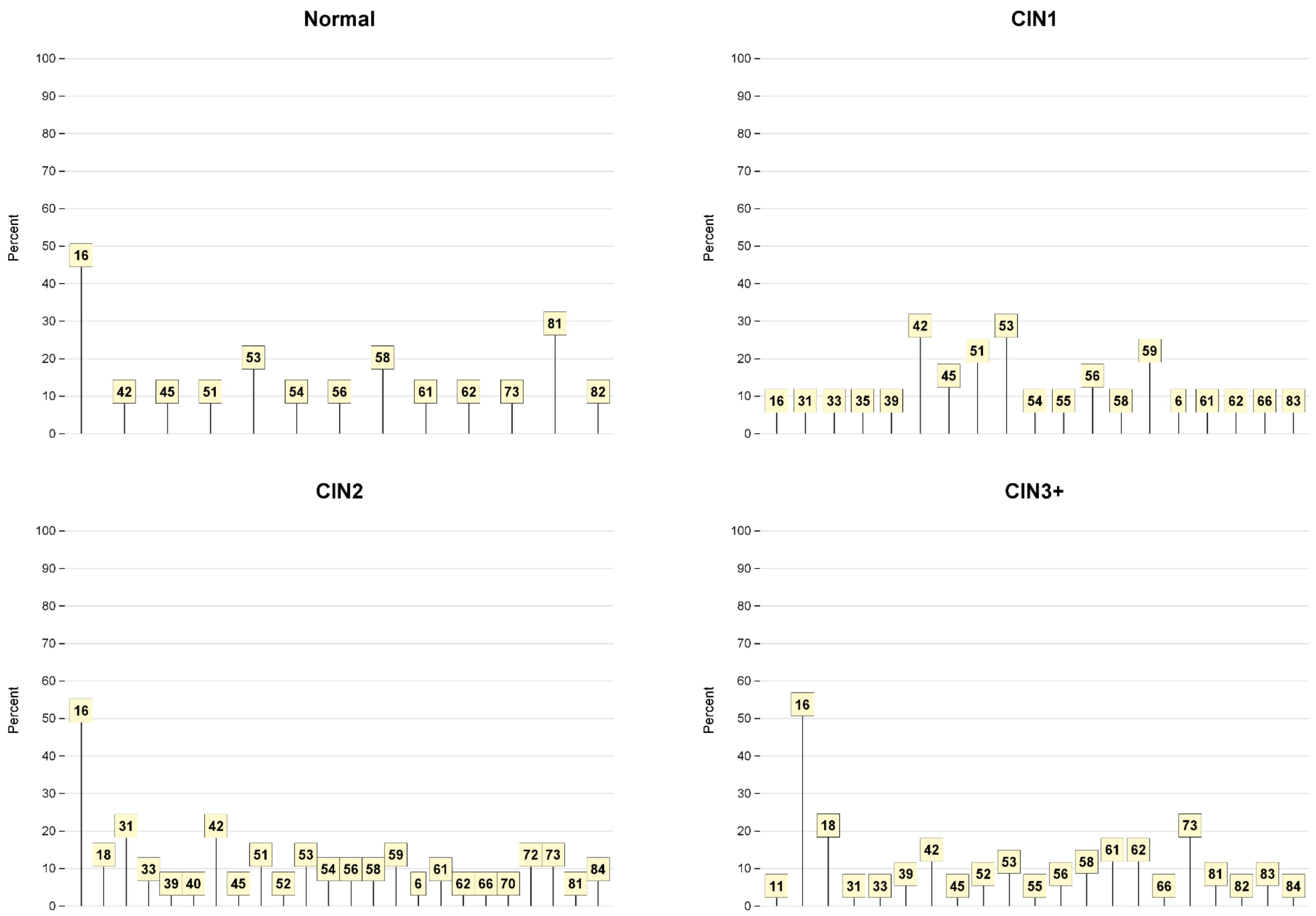

Within square numbers identify the genotype, 16 = HPV 16, 108 = HPV CP6108 etc; Minimun Sample Size N = 10;

Figure S4. HPV genotypes frequency distribution needle plots by histology for LR Richness ≥ 2

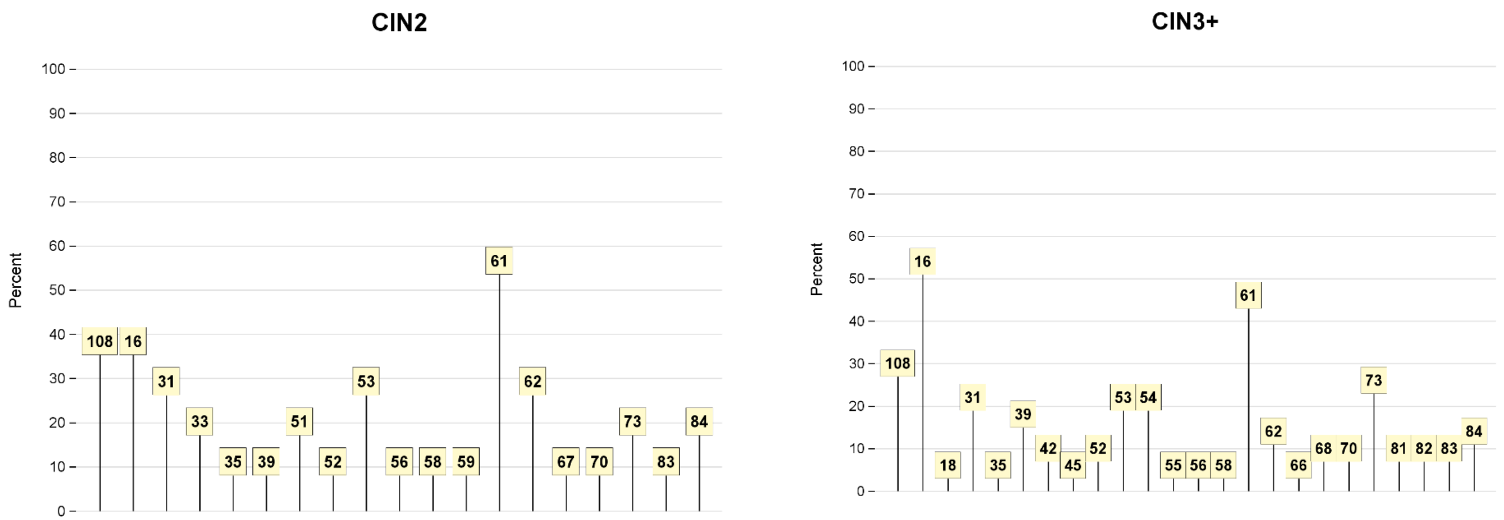

Within square numbers identify the genotype, 16 = HPV 16, 108 = HPV CP6108 etc; Minimun Sample Size N = 10;
